# Self-perception of dietary quality and adherence to food groups dietary recommendations among Mexican adults

**DOI:** 10.1186/s12937-020-00573-5

**Published:** 2020-06-22

**Authors:** Carolina Batis, Analí Castellanos-Gutiérrez, Tania C. Aburto, Alejandra Jiménez-Aguilar, Juan A. Rivera, Ivonne Ramírez-Silva

**Affiliations:** 1 CONACYT – Health and Nutrition Research Center, National Institute of Public Health, Av. Universidad No. 655 Colonia Santa María Ahuacatitlán, C.P, 62100 Cuernavaca, Morelos Mexico; 2 Health and Nutrition Research Center, National Institute of Public Health, Av. Universidad No. 655 Colonia Santa María Ahuacatitlán, C.P, 62100 Cuernavaca, Morelos Mexico; 3 Department of Nutrition, University of North Carolina at Chapel Hill, 123 W. Franklin St, Chapel Hill, NC 27516 USA

**Keywords:** Perception, Diet quality, Diet, Mexican

## Abstract

**Background:**

Mexicans’ adherence to food group’s dietary recommendations is low and an inaccurate self-perception of dietary quality might perpetuate this low adherence. Our aim was to compare the intake and the adherence to the dietary recommendations for several food groups, subgroups, and to an overall Mexican Diet Quality Index (MxDQI), among those that perceived their diet as healthy vs. those that did not.

**Methods:**

We analyzed data from 989 subjects 20–59 y old from the nationally representative Mexican National Health and Nutrition Survey 2016. Dietary intake was collected with one 24-h recall and a repeated recall in 82 subjects. Self-perception of dietary quality was evaluated with the following question “Do you consider that your diet is healthy? (yes/no)”. We used the National Cancer Institute method to estimate the usual intake. We compared the mean intake adjusted by sociodemographic variables and the percentage of adherence according to the self-perception of dietary quality among the whole sample and in sociodemographic subpopulations.

**Results:**

Sixty percent perceived their diet as healthy, and their adherence to recommendations was low [20% for fruits and vegetables, < 8% for legumes, seafood and SSBs, and ~ 50% for processed meats and high in saturated fat and/or added sugar (HSFAS) products]. The mean number of recommendations they met was 2.8 (out of 7) vs. 2.6 among the rest of the population (*p* > 0.05), and the MxDQI score was 40 vs. 37 (out of 100 points). The only food groups and subgroups with a statistically significant difference between those that perceived their diet as healthy vs. unhealthy were fruits [38 g/d (95% CI 3, 73)], fruit juices [27 g/d (95% CI 2, 52)], industrialized SSBs [− 35 kcal/d (− 70, − 1)] and salty snacks [− 40 kcal/d (− 79, − 1)]. Other differences were small or inconsistent across subgroups of the population.

**Conclusions:**

Those that perceived their diet as healthy only had a slightly healthier diet than the rest of the population, moreover, their adherence to recommendations was very low. Hence, it is necessary to improve their nutrition knowledge.

## Background

Obesity and its associated comorbidities are public health concerns of top-level priority in Mexico. The reported prevalence of overweight and obesity in Mexican adults has risen from 56% in the year 2000 [1] to 72.5% in 2016. Accordingly, the prevalence of diseases directly related to obesity has significantly increased over the past decades, with now more than 25% of adults diagnosed with high blood pressure and 9.4% with diabetes mellitus [2]. Dietary factors are key contributors to the development of obesity and chronic diseases. According to the Global Burden of Disease, Injuries, and Risk Factor study 2013, dietary factors including high intake of sugar-sweetened beverages **(**SSBs), processed meat, and a low intake of whole grains are three of the ten leading risk factors for disability-adjusted life years in Mexican men and women [3]. Despite the high disease burden attributable to dietary factors, in Mexico, adherence to dietary recommendations for food groups is low. In 2012 only 7 to 14% of the population age groups reached the recommended intake for fruits and vegetables, 0.9 to 4% for legumes, while 78 to 90% exceeded the recommendation for the intake of SSBs, 50 to 90% for processed meat, and 58 to 86% for foods high in saturated fat and/or added sugar (HSFAS) [4]. Thus, improving dietary intake has been at the forefront of the national nutritional agenda in the past years [5].

Although there is wide recognition of the influence of environmental determinants in these efforts, changes also have to occur at the individual level. Perceived diet quality is one psychosocial factor that could influence dietary intake, if the perception is inaccurate it could perpetuate poor dietary habits [6]. Previous studies from the Netherlands and the USA found that perception of the healthfulness of dietary intake was unrealistic. For instance a third of the subjects underestimated their fat intake, or perceived their fat intake as “about right” when it was high [7, 8]; 30–38% had a misconception of their vegetables and fruit intake, either they perceived it as sufficient or insufficient when it was not the case [9]; and 40% perceived their diet healthier than what it was objectively according to the Healthy Eating Index [10]. If individuals perceive their dietary intake to be of higher quality than what it actually is, their intention to improve it will be limited [11]. Hence, it is important to assess the accuracy of perceived dietary quality by comparing it with objectively measured dietary quality, and this has not been evaluated in the Mexican population.

The most recent Mexican National Health and Nutrition (ENSANUT) conducted in 2016 included, for the first time in a Mexican national survey, a questionnaire about self-perception of dietary intake. Therefore, our aim was to compare the intake and the adherence to the dietary recommendations for several food groups and subgroups, according to dietary quality perception, in the whole sample and in sociodemographic subpopulations.

## Methods

### Study population

We used data obtained from the ENSANUT 2016, a population-based multistage probabilistic survey representative of the Mexican population at the national, regional and state levels, for urban and rural areas [12]. Information from 29,975 individuals was obtained through face-to-face interviews conducted between May and October 2016 by trained personnel to members of 9474 households.

Dietary intake data were collected on a random subsample of individuals of all ages (*n* = 4188), whereas the questionnaire of self-perception of dietary quality was collected among adults aged 20 to 59 years (*n* = 6550). Our analytical sample included male and non-pregnant non-lactating female adults 20–59 y old with available 24-h recall (*n* = 1023) who completed and gave a valid answer in the dietary quality perception question (*n* = 989). Informed consent was obtained from each subject. The survey protocol was reviewed and approved by the Ethics Committee of the Mexican National Institute of Public Health.

### Dietary data collection and dietary recommendations

Dietary information was collected using 24-h dietary recalls with a five-step multiple-pass method developed by the United States Department of Agriculture and adapted to the Mexican context [13]. Interviewers used scales (if a food similar to the one consumed was available in the household) or common household measuring items such as spoons and cups to estimate portion sizes. The second dietary recall was collected in-person on a subsample on a non-consecutive day after the first recall. Both first and second 24-h recalls were conducted between Monday and Sunday.

We used the same food groups and dietary recommendations that were previously used in a Mexican nationally survey [4]. The food groups were created by a team of three dietitians with a master’s degree. These seven food groups (fruits and vegetables, legumes, seafood, red meats, processed meat, SSBs, and HSFAS products) are not comprehensive of the total diet, as we wanted to analyze only groups that are clearly encouraged or discouraged by current dietary recommendations. Food group recommendations were primarily based on the Mexican Dietary Guidelines [14] and complemented with other international recommendations specific cutoff points [World Health Organization [15] for fruits and vegetables, World Cancer Research Fund/American Institute of Cancer Research [16] for red meat, American Heart Association [17] for processed meats and SSBs, and Dietary Guidelines for Americans 2010 [18] for seafood]. We defined HSFAS products as salty snacks, desserts, sugars, and cereals with > 13% of saturated fat and/or 13% of added sugar, and considered < 10% of energy intake from HSFAS as the recommended intake. The 13% cutoff point was based on the International Choices Program [19], and the < 10% recommendation was selected because we estimated, based on the average intake of our population, that < 10% was compatible with avoiding excessive intake of saturated fat and added sugar [15]. More details on the description of the food groups and a summary of the recommendations used are described in Table 1.

**Table 1:** Summary of recommendations used and description of food groups

Food group	Mexican Dietary Guidelines (MDG)		Recommendation used	Food group description
Fruits and vegetables	6 servings for a 2000-kcal/d (about ≥400 g/d)		≥400 g/d (World Health Organization)	Fresh, frozen, canned, and dried fruit and vegetables, including 100% fruit juices, not including potatoes or avocado.
Legumes	2 servings/d for a 2000-kcal/d		≥2 servings/d (MDG, one serving is 120 kcal or ~ 125 mL of cooked legumes)	Beans, lentils, chickpeas
Seafood		Eat frequently	≥35 g/d (American Heart Association)	Fish and shellfish
Red meat		Limit to < 70 g/d	< 70 g/d (World Cancer Research Fund; grams are of cooked weight)	Beef, pork, lamb, and goat, including that contained in processed meat
Processed meats		Consume the least possible	< 8.6 g/d (American Heart Association)	Meat preserved by smoking, curing, or salting or the addition of chemical preservatives (sausage, ham, dried meat)
SSBs	Limit the intake of foods and beverages with high content of sugar, salt, and fat. Added sugars should not exceed 10% of total energy intake		< 60 kcal/d (American Heart Association)	Non–milk-based caloric beverages: industrialized (soft drinks, fruit drinks, sports drinks, energy drinks, fruit juices/nectars), home-made [coffee/tea with sugar, “agua fresca” (home-made fruit drink)]
HSFAS products			< 200 kcal/d (author’s own estimation of allowable intake to comply with < 10% of added sugar and saturated fat World Health Organization recommendation)	Baked goods (cookies, granola bars, pastries), breakfast cereals, salty snacks (potato chips, tortilla/corn chips, cracker nuts, cheese puffs), candies (chocolate, chewing gum, desserts (ice-cream, gelatin, pudding, ice pop), sugar and sweeteners (white/brown sugar, honey, syrup, chocolate powder) with > 13% of saturated fat and/or > 13% of added sugar.
**Dietary indices**	**Description**			
Mexican Diet Quality Index (MxDQI)	Minimum and maximum points for each component (total range 0–100): Vegetables 0 (0 servings) to 10 (≥ 3 servings); whole fruit 0 (0 servings) to 10 (≥ 3 servings); whole grains 0 (0 servings) to 5 (≥ 3 servings); legumes 0 (0 servings) to 10 (≥ 2 servings); seafood, poultry or eggs 0 (< 1 serving) to 5 (≥ 2 servings); low-fat dairy 0 (0 servings) to 5 (≥ 3.5 servings); polyunsaturated fat 0 (< 6% of total energy intake) to 5 (> 10% of total energy intake); 100% fruit juice 0 (> 250 ml) to 5 (≤125 ml); refined grains 0 (> 3 servings) to 5 (≤1 servings); red and processed meats 0 (> 1.5 servings) to 5 (≤0.5 servings); added sugars 0 (> 10% of total energy intake) to 15 (< 5% of total energy intake); sodium 0 (> 2 g) to 15 (≤1.5 g); saturated fat 0 (> 10% of total energy intake) to (< 7% total energy intake) points. Servings, ml, and g are per 2000 kcal.			
Index of total food group’s recommendation met	For the main 7 food groups (fruits and vegetables, legumes, seafood, red meats, processed meat, SSBs, and HSFAS products), one point for each recommendation met; the range of possible points is 0—7.			

*HSFAS* high saturated fat and/or added sugar; *SSBs* sugar-sweetened beverages

For some food groups, we additionally evaluated the intake of subgroups, fruits and vegetables were subdivided in fruits, vegetables, and 100% fruit juices; SSBs were subdivided in industrialized and home-made SSBs, HSFAS products were subdivided into baked goods and breakfast cereals, salty snacks, candies and desserts, and sugar and sweeteners. Moreover, we estimated two indexes. The first one was the Mexican Dietary Quality Index (MxDQI) developed by López-Olmedo et al [20]. This index is based on the Mexican Dietary Guidelines, it includes 13 components (4 nutrients and 9 food groups) and has a range of 0 to 100 points (see Table 1). For the second index, we added the number of food groups’ recommendations met for the seven main food groups, each recommendation met on that day counted one point, and the possible range was 0 to 7.

We calculated total energy intake and the energy from SSBs and HSFAS products using the Mexican Food Database (BAM, version 1.1) [21], a food composition database compiled by the Mexican National Institute of Public Health with information from Mexican food composition tables, product labels, USDA Standard Reference database, and standard Mexican recipes. In the case of red meat and seafood, we converted all grams that were reported raw to cooked weight using the cooking yield factors from the BAM.

### Self-perception of dietary quality

The ENSANUT 2016 included for the first time in a Mexican national nutrition survey, a “Perception of Obesity, Physical Activity, and Diet Questionnaire”. The aim of the questionnaire was to assess the perception, attitudes, behaviors, barriers, self-efficacy, and knowledge of Mexican adults with regards to obesity, diet, and physical activity. It included 64 closed-ended, multiple-choice questions. For this analysis, we used the question *“Do you consider that your diet is healthy?”* to assess the self-perception of dietary quality. The possible answers were “yes”, “no”, and “I do not know”. We excluded individuals that answered “I do not know”. From here on, we refer to those that answered yes or no, as those that perceive their diet as healthy or unhealthy, respectively. Other questions relevant to this analysis were the following: Do you currently consume ≥5 fruits and vegetables per day? (“yes”, “no”, and “I do not know”); Do you think that SSBs are healthy? (“strongly agree”, “agree”, “disagree”, “strongly disagree”, and “I do not know”).

### Sociodemographic characteristics

Age was categorized into four groups: 20–29, 30–39, 40–49, and 50–59 years old. Socioeconomic status (SES) was determined based on an index created using information that included the household’s assets. Rural areas were defined as areas with less than 2500 inhabitants. Education level was categorized as low (0–6 years of schooling), medium (7–12 years of schooling), and high (> 12 years of schooling). Weight and height (to estimate BMI) were measured by trained personnel.

### Statistical analysis

We estimated the distribution of sociodemographic characteristics by self-perception of dietary quality and compared them with chi-square test. Moreover, we estimated the proportion of subjects that perceived they consume ≥5 fruits and vegetables per day and the proportion that disagreed that SSBs are healthy, according to their overall perception of dietary quality.

Then, we described and compared the intake and adherence to dietary recommendations for the main seven food groups by diet quality perception. We used the National Cancer Institute (NCI) method for episodically consumed foods to estimate the usual intake of food groups consumed [22]. Briefly, this method fits a mixed logistic regression model for the probability of consumption among all subjects, and a mixed linear regression model for the amount consumed only among consumers. For the linear model, a Box-Cox transformation is used and the random effects are separated into person-specific random effects (between-individual variation) and within-individual errors. Based on these models, and excluding the within-individual errors, the distribution of usual intake in the population is estimated. Given that a large sample size with consumption in the two 24-h recalls of the analyzed food groups is needed to obtain stable estimates, we included all subjects with available 24-h recall in the ENSANUT 2016, regardless of the age or completion of the perception questionnaire (*n* = 4188 with one 24-h recall, and *n* = 286 with a second 24-h recall). We included an indicator variable to estimate the distribution of usual intake by subgroup of subjects (i.e., adults 20—59 y old that perceived diet as healthy, adults 20—59 y old perceived diet as unhealthy, and all others age groups) and report only the findings for adults 20—59 y old. We also included in the models the 24-h recall sequence (1st or 2nd) and day of recall (weekend or weekday), so that the distribution could be estimated assuming all recalls were the first recall, and with a 3/4 weekend/weekday ratio. We included the survey weights in the estimation. Based on the estimated usual intake distributions with the NCI method, we present, by dietary quality perception: 1) the median and the 25 and 75 percentiles of the distribution, 2) a graphical representations of the whole distribution, and 3) the % of the population adhering to dietary recommendations (i.e., percentage of subjects above the minimum recommended amounts for healthy food groups, and the percentage of subjects below the maximum recommended amount for unhealthy food groups). This analysis was conducted in SAS 9.4 (SAS Institute Inc. Cary, NC).

Additionally, to conduct statistical comparisons, we used one 24-h recall to estimate the adjusted mean of the seven food groups and subgroups among those that perceived their diet as healthy or unhealthy. We run linear regression models with each food group as the dependent variable, diet quality perception as the independent variable, and sex, age group, rural/urban area, SES, education level, BMI, and the geographical region as covariates. From these models, we obtained the adjusted mean intake among those that perceived their diet as healthy or unhealthy (*margins* command in STATA) and the adjusted difference [β (95% CI)] between them. To understand if the later analyses differed by socioeconomic characteristic, for the main seven food groups, we repeated the same models but adding an interaction term between diet quality perception and each sociodemographic characteristic (we run separate models for each sociodemographic variable). We present the predicted difference [β (95% CI)] in each sociodemographic stratum, and indicate when the interaction term had a *p*-value < 0.10. We performed a “chunk” test to jointly test all the interaction terms in the case of sociodemographic variables with dummies. This analysis using only one 24-h recall was conducted in STATA 14 [StataCorp, College Station, TX]) with the survey prefix command (svy) to account for the complex design.

### Sensitivity analysis

Considering that the question *“Do you consider that your diet is healthy? (yes/no)”* is very subjective and prone to social-desirability bias (e.g., subjects were embarrassed to admit that their diet is not healthy) we conducted two sensitivity analyses. In the first one, we excluded under reporters of energy intake, assuming these subjects might be more prone to social-desirability bias with regards to their dietary intake. We followed the methodology of Huang et al. [23] and excluded subjects with ≤56% according to the following formula: (reported Energy Intake/predicted Energy Requirements) * 100. We estimated the Energy Requirements with the Institute of Medicine equations [24], and we assumed a low physical activity level among men (1.11 for nonobese and 1.12 for obese), and a sedentary level among women (1.0 for nonobese and obese) [13]. The ≤56% cutoff point was equivalent to < 2 Standard Deviations (SD) in Huang et al. sample, and we used this instead of estimating our own cutoff point because we lacked the intraindividual variation in energy intake needed for this calculation. For the second sensitivity analysis, we used the question “Do you currently consume ≥5 fruits and vegetables per day?” as a proxy of perception of a healthy diet. Assuming this question is more objective, and that healthiness is frequently associated with the intake of fruits and vegetables as previous studies have shown [25–27].

## Results

Sixty percent of adults 20–59 y old perceived their diet as healthy (e.g., answered “yes” when asked: *“Do you consider that your diet is healthy?”)*. The distribution of sociodemographic characteristics by self-perception of dietary quality was similar. The only differences were in the distribution of residence area, and BMI. The proportion of subjects living in rural areas and with normal BMI was higher among those that perceived their diet as healthy in comparison to those that perceived it as unhealthy (Table 2). The proportion of subjects that perceived that they consume ≥5 fruits and vegetables was 34% among those that perceive their diet as healthy, and 19% among those that perceived it as unhealthy. The proportion of subjects that agreed that SSBs are healthy was ~ 5% among both dietary quality perception groups (Table 3).

**Table 2:** Sample characteristics by self-perception of dietary quality

	All	Perceived their diet as healthy^1^	Perceived their diet as unhealthy	*p-value*^2^
	%			
All	100	60.1	39.9	
Sex				
Men	46.2	45.8	46.8	0.88
Women	53.8	54.2	53.2	
Age group				
20–29 y	24.2	19.9	30.7	0.16
30–39 y	27.8	30.6	23.6	
40–49 y	30.2	30.5	29.8	
50–59 y	17.8	19.1	16.0	
Residence area				
Rural	23.0	28.4	14.9	0.00
Urban	77.0	71.6	85.1	
SES				
Low	18.2	20.9	14.2	0.22
Medium	26.1	25.8	26.5	
High	55.8	53.4	59.4	
Education				
Low	26.2	30.2	20.1	0.15
Medium	50.1	45.7	56.8	
High	23.7	24.2	23.1	
BMI				
Normal	23.1	26.2	18.6	0.03
Overweight	36.0	39.3	31.0	
Obesity	40.9	34.6	50.4	
Region				
North	19.7	23.5	14.1	0.21
Center	32.1	30.1	35.0	
Mexico City	18.0	15.9	21.1	
South	30.2	30.5	29.8	

^1^Answered *“yes”* from *“yes”* or *“no”* options, when asked: *“Do you consider that your diet is healthy?”*

^2^Chi-square test comparing distribution of characteristics by self-perction

**Table 3:** Perception and 1-day and usual intake by self-perception of dietary quality

	Perceived as healthy^1^	Perceived as unhealthy	Difference between perceived as healthy^1^ vs. unhealthy	Perceived as healthy^1^	Perceived as unhealthy	Perceived as healthy^1^	Perceived as unhealthy
	*Mean or % ± SE*^*2*^		*β (95% CI)*^*2*^	*% Adhering to recommendation*		*Median (p 25, p50)*	
**Perception questions**							
Currently consume ≥5 fruits and vegetables, %	34 ± 4	18 ± 4	15 (4, 27)				
Agree SSBs are healthy, %	7 ± 2	4 ± 1	2 (−2, 6)				
**24-h dietary recall**	**1-day**			**Usual intake (NCI method)**			
Fruits and vegetables, g/d	288 ± 20	215 ± 20	**74 (16, 132)**	19.9	13.4	278 (203, 373)	243 (176, 331)
Vegetables	136 ± 10	127 ± 12	9 (−23, 41)				
Fruits	121 ± 12	84 ± 12	**38 (3, 73)**				
100% fruit juices	31 ± 13	4 ± 4	**27 (2, 52)**				
Legumes, servings/d	0.54 ± 0.05	0.52 ± 0.07	.02 (−0.15, 0.19)	4.3	3.2	0.52 (0.27, 0.92)	0.46 (0.24, 0.82)
Seafood, g/d	14 ± 5	7 ± 3	8 (−4, 20)	3.9	2.2	3 (1, 8)	2 (1, 6)
Red meat, g/d	42 ± 7	35 ± 5	7 (−13, 26)	86.5	81.7	31 (16, 53)	38 (21, 61)
Processed meats, g/d	17 ± 3	12 ± 3	5 (−2, 13)	50.6	55.2	8 (4, 17)	7 (4, 15)
Sugar-sweetened beverages (SSBs), kcal/d	190 ± 15	221 ± 15	−31 (−75, 13)	7.9	2.2	147 (99, 204)	254 (168, 360)
Industrialized	116 ± 10	151 ± 14	**−35 (−70, −1)**				
Home-made	74 ± 10	70 ± 10	4 (−22, 31)				
High saturated fat and/or added sugar (HSFAS) products, kcal/d	233 ± 26	272 ± 30	−39 (− 124, 46)	55.8	46.8	179 (100, 285)	213 (120, 334)
Baked goods and breakfast cereals	151 ± 19	131 ± 17	19 (−28, 67)				
Salty snacks	21 ± 6	61 ± 19	**−40 (−79, −1)**				
Candies and desserts	16 ± 3	36 ± 14	−20 (−49, 9)				
Sugar and sweeteners	45 ± 17	44 ± 12	−1 (−43, 46)				
Total energy, kcal/d	1921 ± 60	1894 ± 60	27 (− 128, 181)				
Mexican Diet Quality Index (MxDQI)	40 ± 1	37 ± 1	**3 (1, 6)**				
Index of total food group’s recommendation met	2.80 ± 0.08	2.65 ± 0.08	0.14 (−0.08, 0.37)				

^1^answered *“yes”* from *“yes”* or *“no”* options, when asked: *“Do you consider that your diet is healthy?”*

^2^Adjusted by sex, age group, residence area, socioeconomic status, education level, BMI, and geographical region. Bold numbers have a *p* < 0.05

**Fig. 1:**
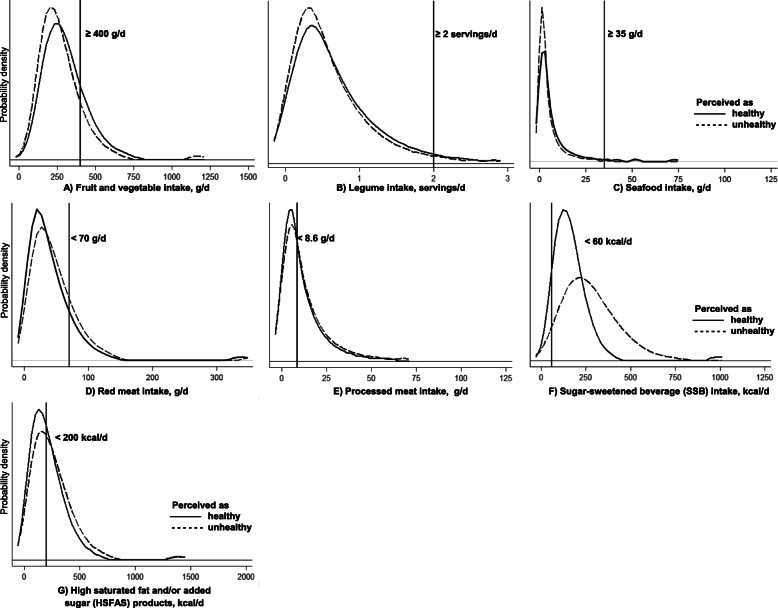
Dietary recommendations and usual intake distributions for food groups by self-perception of dietary quality.^1 1^Usual intakes were estimated with the National Cancer Institute method for episodically consumed foods [22]

The adherence to dietary recommendations was very low for all the main seven food groups (except red meat), even among those that perceived their diet as healthy (Table 3 and Fig. 1). For instance, among those that perceived their diet as healthy only 20% reached the recommended intake for fruits and vegetables, 4% for legumes, and seafood; whereas only 51% did not exceed the recommended upper level for processed meats, 8% for SSBs, and 56% for HSFAS products. When comparing the adherence to recommendations by diet quality perception, we found that those that perceived their diet as healthy shown a better adherence to the recommendations of fruits and vegetables, red meat, SSBs, and HSFAS products. While they had a worse adherence to the recommendations of processed meats, and a similar adherence to the recommendations of legumes and seafood. However, in terms of statistical significance, when comparing the adjusted mean intake, only the intake of fruits and vegetables was statistically significant (*p* < 0.05) different among dietary quality perception groups. For subgroups, we found that those that perceived their diet as healthy had a statistically significant (*p* < 0.05) higher intake of fruits and fruit juices, and a lower intake of industrialized SSBs, and salty snacks, compared to those that perceived their diet as unhealthy. Total energy intake was very similar across dietary quality perception groups. Regarding dietary indexes, the points for the MxDQI were statistically significant (*p* < 0.05) higher among those that perceived their diet as healthy, but they only had 3 more points while the range of the index is 0–100. The total number of food group recommendations’ met in 1-day was 2.8 (out of 7) among those that perceived their diet as healthy and 2.6 among those that perceived it as unhealthy.

**Fig. 2:**
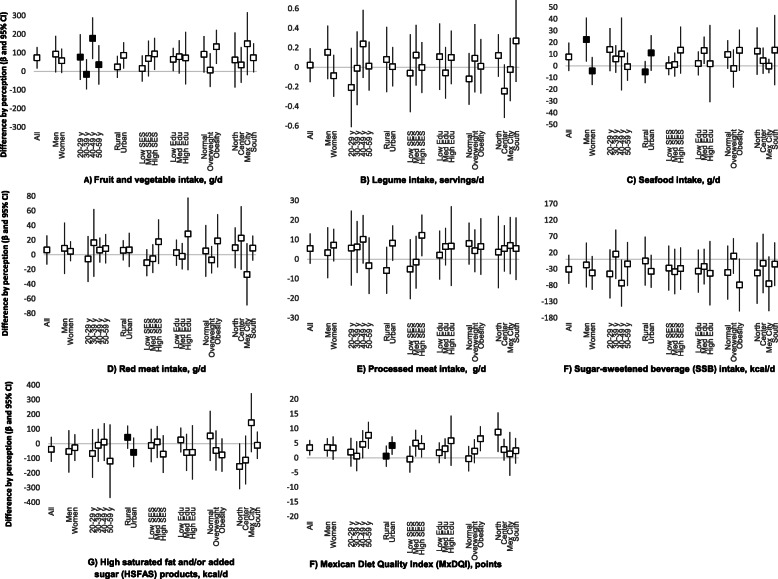
Differences between perceived as healthy vs. unhealthy in the intake of food groups.^1 1^Differences (β and 95% CI) were obtained from models with an interaction term for subpopulations and adjusted by sex, age group, residence area, socioeconomic status, education level, BMI, and geographical region. Point estimates are black if the interaction term had a *p* < 0.10

In Fig. 2 we present the difference (and 95% CI) in the mean intake of the main seven food groups and the MxDQI between those that perceived their diet as healthy vs. unhealthy, among the entire sample, by BMI and by sociodemographic subpopulations. A positive difference means that the intake was higher among those that perceived their diet as healthy, and the units of the difference depend on the food group [e.g., for fruits and vegetables among all the population the β (95% CI) was 74 (16, 132); meaning that those perceiving their diet as healthy consumed 74 g/d more fruits and vegetables than those perceiving their diet as unhealthy]. In most cases, within each subpopulation the difference was not statistically significant, as the 95% included the zero. Moreover, for most food groups the point estimate of the difference was close to zero in all subpopulations; only for fruits and vegetables, SSBs, and the MxDQI, the differences were predominantly positive or negative. We found few statistically significant interactions (*p* < 0.10) by sociodemographic characteristics, which are highlighted in black in the figure. For fruit and vegetables, the interaction between perception and age group was statistically significant (those 30–39 y old tended towards a negative difference whereas other age groups tended towards a positive one). Interaction between perception and sex, and between perception and urban/rural was significant for seafood intake (women and rural, tended towards negative differences, whereas their counterpart tended towards a positive one). Interaction between perception and urban/rural was also significant for HSFAS products intake (urban population tended towards a negative effect, whereas rural tended towards a positive one) and for the MxDQI (the positive effect was stronger among urban than rural populations). Moreover, although there was no statically significant interaction, some subgroups of the population had a strong association between intake and perception that was contrary to the recommendation. For instance, the difference was negative for legume intake among the Center region, and the difference was positive for processed meats intake among women, 40—49 y olds and high SES.

We present the results of the sensitivity analyses on the **Supplemental Table** **1****.** We found that in both cases (excluding under reporters of energy intake or using the perceived intake of fruits and vegetables as a proxy of perception of a healthy diet) the results were similar to our main findings, but the effect was stronger.

## Discussion

In this analysis, we assessed for the first time in a nationally representative survey in Mexico the self-perception of dietary quality and compared it against the intake and adherence to dietary recommendations for several food groups. We found, that despite the high prevalence of overweight and obesity and the documented low dietary quality of the Mexican population, the majority (60%) of the adults perceived their diet as healthy (e.g., answered “yes” when asked: *“Do you consider that your diet is healthy?”)*. The adherence to recommendations was very low for most food groups and the intake of those that perceived their diet as healthy was not different (*p* > 0.05) than the intake of those that perceived their diet as unhealthy. The only exceptions were a higher (*p* < 0.05) intake of fruits, and a lower intake of industrialized SSBs, and salty snacks. Thus, it appears that only these three food groups are correctly perceived as healthy and unhealthy, respectively. The MxDQI score was higher (*p* < 0.05) among those that perceived their diet as healthy, but only by 3 points (from a 0–100 index).

Studies of self-perception of dietary quality and actual dietary intake are of importance because they can potentially show the disconnect between these in the population. This disconnect referred as ‘optimistic bias’ might be an obstacle to improving diet quality, given that individuals who consider their diet is already healthy, might not see the need to improve it [28]. Similar to what we observed in the Mexican population, studies in the USA have shown that people tend to overrate their dietary quality, perceiving or grading their diets as healthier than what they actually are. For instance, self-perception of diet quality and calculated diet quality assessed with the Healthy Eating Index showed that 40% of respondents perceived their diet to be healthier than it actually was [10]. Similarly, Americans that perceived their diet quality as being high had a DASH index score of 3 out of a maximum of 9 [6]. In our study, the score was 40 out of 100 possible points for the MxDQI. Still, in previous studies, individuals that perceived their diet quality as high or good had a higher diet quality (assessed with diet indexes) than those who perceived their diet quality as low or poor [6, 26]. Moreover, and somewhat similar to what we observed, this difference appears to be driven by fruit, vegetable, and empty calories intake [26]. Sharif, et al. also observed higher fruit and vegetable intake, and lower soda intake among Latinos that self-rated their diet quality as good compared to those who self-rated it as poor. Nonetheless, and again, similar to our findings, soda intake was high in both groups [29].

Our results suggest that Mexican adults are aware that fruits are healthy, and industrialized SSBs and salty snacks unhealthy. Whereas for other food groups the population might not even be aware if these are healthy or not. It should be noted that all the recommendations assessed are part of the official Mexican nutrition education documents, which are the basis for nutrition education in the health-care settings and in schools [14, 30]. However, many recommendations are qualitative (i.e., “limit/promote/eat a lot”), and are not promoted nationally. Hence, according to our results promotion for other food groups is needed, and quantitative guidance might be warranted, as a “limit/promote” recommendation is not getting through the population to the level of intake nutrition research has established.

With regards to the food groups that were correctly associated with a healthy/unhealthy diet; for years it has been recommended the intake of fruits and vegetables to the population. One example of a mass-media education message is the Ministry of Health’s requirement of the inclusion of a healthy legend such as “eat fruits and vegetables”, in media advertisements of foods and beverages of low nutritional quality [31], and the 5-a-day campaign has also been widespread. Hence, it was not surprising that the intake of fruits was higher among those that perceived their diet as healthy. On the other hand, we did not find differences in vegetables. Usually, vegetables are consumed as part of preparations and not in isolation, this might “mask” vegetables and make individuals less aware of their intake.

In the case of industrialized SSBs, recently, a set of policies aimed at reducing the intake of industrialized SSB’s including banning them from schools, restricting advertising, and imposing taxes to them [32–34], seem to have had effects on public awareness of their negative health effects. For instance, as part of the SSB’s tax advocacy in Mexico, the civil society launched media campaigns educating the population about the health harms of SSBs [35]. According to a national poll, from 2013 to 2014 the percentage of subjects that agreed that SSBs were a risk factor for obesity, caries, hypertension, and cancer increased 5 to 10 percentage points [36]. Moreover, the implementation of the SSB tax itself can have a “signaling effect” and inform the population about the health risks associated with the intake of SSBs. Álvarez-Sánchez et al., based on the perception questionnaire of the ENSANUT 2016, documented that adults that were aware of the implementation of the SSB tax consumed fewer SSBs in comparison to those that were not aware [37]. All these policies have focused on industrialized SSBs and correspondingly we found that only industrialized SSBs were consumed in lower amounts by those that perceived their diet as healthy, whereas the intake of home-made SSBs was not different. Industrialized SSBs are more energy-dense than homemade SSBs, and in the Mexican population, these contribute with 90 kcal/d of added sugar in comparison with 55 kcal/d from home-made SSBs [38]. Hence, it is reassuring that the association with the perception of dietary quality is stronger for industrialized SSBs. Yet, the intake of home-made SSBs is also considerable and subjects need to be more aware of their detrimental effects on health.

In the case of HSFAS products that include baked goods, breakfast cereals, salty snacks, candies, desserts, sugar, and sweeteners; only the intake of salty snacks was different by dietary quality perception groups. It is possible that other HSFAS products are either not perceived as discretionary items because they are consumed as part of main meals (baked goods, breakfast cereals, sugar, and sweeteners), or their contribution is smaller and hence not relevant from subjects’ perception (candies and desserts). Whereas it might be that salty snacks, are clearly identified as “unhealthy” snacks and their contribution to intake is notable by subjects’ perception.

The recommendations that we used to assess the dietary quality of our population are based on the Mexican dietary guidelines, which were in turn based on international recommendations for a healthy diet [4, 14]. However, recently, guidelines for a reference diet that in addition to healthy is environmentally sustainable were released by the *EAT-Lancet* Commission [39]. The main difference between the recommendations that we used and the Commission’s recommendations is that the upper limit of red meat is much lower in the latter (70 g/day vs. 14–28 g/day). We found that the 25th percentile of red meat intake was 16 and 21 g/day among dietary quality perception groups, meaning that adherence for the commission’s red meat recommendation would be ~ 25% instead of > 80% as we reported. Yet, the Commission emphasizes that this red meat recommendation should be carefully considered in each context, as there might be subgroups of the populations for which red meat is a nutrient-rich source.

Our study is not without limitations. The disconnect between perception and actual intake could be related to a lack of knowledge of the recommendations, or also to an incorrect assessment of the amounts consumed. In our analysis, we cannot distinguish the sources of this disconnect. An additional questionnaire such as the one designed by Asaad et al., [40] in which the subject is asked about following specific recommendations would have been useful for this purpose. In our survey, this was only inquired for fruits and vegetables. As part of the perception questionnaire individuals were asked: “Do you currently eat at least five fruits or vegetables a day?” and only 34% of those that considered their diet as healthy answered yes. Hence, it is evident that, at least in this case, those that perceived their diet as healthy are unaware that the amount recommended is at least 5 servings of fruits and vegetables. Other sources of disconnect between perception and actual intake could be that the term “healthy diet” is very subjective and unclear to the population, or that subjects are embarrassed to admit that their diet is unhealthy (social-desirability bias). The two sensitivity analyses that we conducted suggest that these two sources of error might be present, as the results were stronger in the sensitivity analyses. Hence, the lack of differences by perception that we found in most food groups, including the Index of total food group’s recommentdation met, might be related to this. Future studies should include questions on what is understood by a healthy diet. Moreover, including a broader range of answering options, such as a five-point scale, instead of “yes” or “no”, might give subjects that do not want to admit their diet is unhealthy a less extreme answer, and hence result in a more informative analysis. Nevertheless, this was the first time that a nationally representative sample with dietary intake assessed was also inquired about their self-perception of dietary quality, which allowed us to gain important insights into the relation between intake and perception in the Mexican population. Moreover, our results are consistent with studies from other populations.

## Conclusions

In sum, to our knowledge, we evaluated for the first time in a nationally representative sample of Mexican adults if there is a disconnect between actual intake and perception. We found that 60% of the population perceived their diet as healthy (e.g., answered “yes” when asked: *“Do you consider that your diet is healthy?”)*, yet among them, the adherence to dietary recommendations and their overall MxDQI score was still very low. Their adherence was only slightly better for fruits, industrialized SSBs, and salty snacks. These results suggest that the population is aware that fruits are healthy and industrialized SSBs and salty snacks are unhealthy but they do not have a good approximation of the recommended amounts. In addition, for the remaining food groups such as vegetables, legumes, seafood, red meat, processed meats, and other HSFAS products (baked goods and breakfast cereals, candies and desserts, sugar and sweeteners) the population is either not even aware if these are healthy or not, or they do not consider their intake as an important indicator of a healthy diet. This study highlights the importance of improving the nutrition education of the Mexican population; a first step towards making healthier food choices is seeing the need for doing so. Moreover, these actions should be accompanied by strengthening the policies aimed at improving the food environment and making the healthy choice the easy one.

## Supplementary information

**Additional file 1: Table S1.** Sensitivity analyses excluding under reporters of total energy intake and comparing the intake according to the perception of consuming ≥5 fruits and vegetables per day.^1^

## Data Availability

The dataset with the perception questionnaire analyzed during the current study is available in the ENSANUT’s repository (https://ensanut.insp.mx/). The 24-h recall data analyzed during the current study is available from the corresponding author on reasonable request.

## Abbreviations

SSBs: Sugar-sweetened beverages
HSFAS: High in saturated fat and/or added sugar
ENSANUT: Mexican National Health and Nutrition
NCI: National Cancer Institute
