# Supplementary material for: Self-perception of dietary quality and adherence to food groups dietary recommendations among Mexican adults
Source: Nutr J. 2020 Jun 22;19:59. doi: 10.1186/s12937-020-00573-5 (PMC7310597; doi:10.1186/s12937-020-00573-5)
Supplement: Supplementary file 1 — Additional file 1: Table S1. Sensitivity analyses excluding under reporters of total energy intake and comparing the intake according to the perception of consuming ≥5 fruits and vegetables per day.1 [file 12937_2020_573_MOESM1_ESM.docx]

| **Supplemental Table 1. Sensitivity analyses excluding under reporters of total energy intake and comparing the intake according to the perception of consuming ≥5 fruits and vegetables per day.^1^** | | | | | | | |
| --- | --- | --- | --- | --- | --- | --- | --- |
|  | Excluding under reporters | | |  | Consume ≥5 fruits and vegetables/day | | |
|  | Perceived  as  healthy^2^ | Perceived  as  unhealthy | Difference between  perceived as healthy^2^ vs. unhealthy |  | Yes | No | Difference between  yes vs. no |
| n (%) | 490 (61%) | 272 (39%) |  |  | 254 (29%) | 592 (71%) |  |
|  | *Mean ± SE^3^* | | *β (95% CI)^3^* |  | *Mean ± SE^3^* | | *β (95% CI)^3^* |
| Fruits and vegetables, g/d | 308 ± 23 | 222 ± 24 | **86 (19, 153)** |  | 340 ± 31 | 241 ± 19 | **99 (27, 171)** |
| Vegetables | 149 ± 13 | 138 ± 14 | 11 (-28, 51) |  | 140 ± 14 | 129 ± 11 | 11 (-24, 46) |
| Fruits | 123 ± 14 | 79 ± 13 | **44 (6, 82)** |  | 157 ± 23 | 96 ± 10 | **60 (12, 109)** |
| 100% fruit juices | 36 ± 15 | 6 ± 5 | **31 (2, 60)** |  | 43 ± 26 | 15 ± 6 | 28 (-22, 78) |
| Legumes, servings/d | 0.59 ± 0.07 | 0.53 ± 0.08 | 0.06 (-0.15, 0.28) |  | 0.56 ± 0.09 | 0.52 ± 0.05 | 0.04 (-0.16, 0.25) |
| Seafood, g/d | 15 ± 6 | 6 ± 4 | 8 (-7, 24) |  | 14 ± 5 | 10 ± 4 | 5 (-7, 16) |
| Red meat, g/d | 47 ± 9 | 39 ± 6 | 7 (-18, 31) |  | 36 ± 5 | 41 ± 6 | -6 (-19, 7) |
| Processed meats, g/d | 20 ± 3 | 13 ± 3 | 6 (-3, 16) |  | 14 ± 3 | 16 ± 2 | -2 (-9, 5) |
| Sugar-sweetened beverages (SSBs), kcal/d | 210 ± 18 | 247 ± 17 | -37 (-90, 16) |  | 162 ± 19 | 208 ± 13 | **-47 (-91, -2)** |
| Industrialized | 128 ± 12 | 173 ± 16 | **-45 (-86, -4)** |  | 98 ± 16 | 130 ± 9 | **-32 (-64, -1)** |
| Home-made | 83 ± 12 | 74 ± 11 | 8 (-23, 39) |  | 64 ± 10 | 78 ± 10 | -14 (-42, 13) |
| High saturated fat and/or added sugar (HSFAS) products, kcal/d | 265 ± 31 | 308 ± 35 | -43 (-145, 60) |  | 202 ± 25 | 258 ± 27 | -57 (-132, 19) |
| Baked goods and breakfast cereals | 171 ± 22 | 143 ± 20 | 28 (-28, 85) |  | 145 ± 22 | 149 ± 19 | -4 (-59, 51) |
| Salty snacks | 24 ± 8 | 74 ± 23 | **-50 (-99, -1)** |  | 20 ± 8 | 34 ± 10 | -14 (-37, 9) |
| Candies and desserts | 16 ± 4 | 42 ± 16 | -26 (-63, 10) |  | 18 ± 6 | 19 ± 4 | -1 (-15, 14) |
| Sugar and sweeteners | 54 ± 21 | 48 ± 14 | 5 (-47, 57) |  | 18 ± 10 | 56 ± 18 | -38 (-84, 9) |
| Total energy, kcal/d | 2146 ± 55 | 2090 ± 58 | 56 (-93, 204) |  | 1845 ± 97 | 1917 ± 51 | -72 (-281, 137) |
| Mexican Diet Quality Index (MxDQI) | 40 ± 1 | 36 ± 1 | **3 (1, 6)** |  | 44 ± 2 | 38 ± 1 | **7 (3, 10)** |
| Index of total food group’s recommendation met | 2.68 ± 0.09 | 2.52 ± 0.07 | 0.17 (-0.06, 0.39) |  | 3.05 ± 0.09 | 2.64 ± 0.07 | **0.41 (0.19, 0.64)** |
| ^1^Under reporters were defined as the subjects with ≤56% ([reported Energy Intake/predicted Energy Requirements) * 100].  ^2^Answered *“yes”* from *“yes”* or *“no”* options, when asked: “*Do you consider that your diet is healthy?*  ^3^Adjusted by sex, age group, residence area, socioeconomic status, education level, BMI, and geographical region. Bold numbers have a p<0.05 | | | | | | | |
